# Evolving Mental Health Dynamics among Medical Students amid COVID-19: A Comparative Analysis of Stress, Depression, and Alcohol Use among Medical Students

**DOI:** 10.3390/medicina59101854

**Published:** 2023-10-18

**Authors:** Codruța Alina Popescu, Ana Maria Tegzeșiu, Soimița Mihaela Suciu, Bogdan Florin Covaliu, Sebastian Mihai Armean, Tudor Adrian Uță, Alexandru Constantin Sîrbu

**Affiliations:** 1 Department of Human Science, University of Medicine and Pharmacy “Iuliu Hatieganu”, 400174 Cluj-Napoca, Romania; cpopescu@umfcluj.ro; 2 Counseling Center, University of Medicine and Pharmacy “Iuliu Hatieganu”, 400012 Cluj-Napoca, Romania; 3 Department of Physiology, University of Medicine and Pharmacy “Iuliu Hatieganu”, 400012 Cluj-Napoca, Romania; ssuciu@yahoo.com; 4 Department of Community Medicine, Public Health and Management, University of Medicine and Pharmacy “Iuliu Hatieganu”, 400012 Cluj-Napoca, Romania; covaliu.bogdan@umfcluj.ro; 5 Department of Pharmacology Toxicology and Clinical Pharmacology, University of Medicine and Pharmacy “Iuliu Hatieganu”, 400012 Cluj-Napoca, Romania; Sebastian.Armean@umfcluj.ro (S.M.A.); sirbu.alexandruconstantin@elearn.umfcluj.ro (A.C.S.); 6 Infectious Diseases Hospital, 400348 Cluj-Napoca, Romania; tudor.adri.uta@elearn.umfcluj.ro

**Keywords:** medical students, COVID-19, mental health, academic stress, MSSQ, alcohol use, depression, gender disparities, substance use

## Abstract

*Background and Objectives*: The ongoing COVID-19 pandemic has posed an array of new challenges for medical students worldwide. Amidst academic rigors, students are confronted with unique stressors, potentially affecting their mental health and substance use. This study aimed to investigate the multifaceted effects of depression, alcohol use, and stress on medical students and discern how these factors have been amplified by the pandemic’s circumstances, and to identify predictors of mental distress during the COVID-19 pandemic period. *Materials and Methods*: Two online anonymous and cross-sectional surveys were conducted at the University of Medicine and Pharmacy “Iuliu Hatieganu” in Romania among medical students in 2018 and in 2022. Data were gathered via online questionnaires distributed through closed student groups on social media platforms, with a total of 1061 participants, to investigate stress, depression, alcohol and drug use, and the impact of the pandemic and online education on mental health, maintaining anonymity and ethical approval. The Medical Student Stress Questionnaire (MSSQ) was employed to measure different aspects of stress, the Beck Depression Inventory (BDI) provided insights into the participants’ depressive symptoms, and for the assessment of alcohol consumption habits, the Alcohol Use Disorders Identification Test (AUDIT) was utilized. *Results*: Our findings showed a significant decrease in mean depression scores (13.81 vs. 11.56, with *p* < 0.001) from 2018 to 2022. In 2018, students scored significantly higher in the overall stress-related domains. Additionally, being female, facing financial constraints, and being in pre-clinical years emerged as predictors of heightened academic-related stress and depressive symptoms. Students who had experienced the loss of a family member due to COVID-19 exhibited a statistically significant rise in their average BDI score and current anxiety levels. *Conclusions*: The pandemic, despite introducing new stressors, may have indirectly fostered an increased focus on students’ mental health, leading to more refined support mechanisms. Specialized interventions, taking into account gender and financial problems, are needed to address the multifaceted challenges faced by medical students. Our study highlights the ongoing need to nurture both the academic and emotional strength of future medical practitioners.

## 1. Introduction

The COVID-19 pandemic, triggered by the Severe Acute Respiratory Syndrome Coronavirus-2 (SARS-CoV-2), was initially detected in Wuhan, China, in December 2019. It gradually disseminated across the globe, leading the World Health Organization to proclaim it a global pandemic in March 2020 [1]. This marked the beginning of a period that would affect the lives of many for several months to come [2].

The authorities in Romania responded to the outbreak by implementing a series of preventative measures in a phased manner. These measures included a mandatory 14-day institutional quarantine for individuals traveling from affected regions in Italy starting on 21 February, as well as a ban on public gatherings and closure of schools between 8–13 March. On 16 March, a 30-day state of emergency was declared, followed by a national lockdown on 24 March. The state of emergency was extended by another 30 days on 14 April. In response to the significant increase in the number of new COVID-19 cases and hospitalizations in March 2020, Romania implemented stricter safety measures, including remote work recommendations, isolation, social distancing, and the closure of educational institutions, workplaces, and entertainment venues.

A report suggests that the social inconveniences mentioned earlier can cause emotional outbursts, irritation, and disruptions in eating and sleeping patterns, ultimately leading to mood deterioration and frustration [3]. The sudden transition to distance learning, uncertainty surrounding education, and an unstable job market make students particularly susceptible to various stressors [4].

Studies were conducted to examine changes in emotional and stress patterns in college students during the pandemic. We highlight the results from a study that evaluated college students at a medium-sized public research university at the beginning of the pandemic in 2019 and also in 2020 [5]. The study examined the impact of the COVID-19 pandemic on college students’ mental health and behaviors. During the early stages of the pandemic, students reported significantly higher levels of mood disorder symptoms, perceived stress, and alcohol misuse compared to pre-pandemic levels. Greater concern about COVID-19 was associated with more pronounced symptoms. The findings emphasize the need for support and interventions for college students, especially related to alcohol misuse, and provide insights into how resilience and well-being can evolve during a pandemic.

A meta-analysis analyzed the substantial shifts in higher education triggered by the worldwide COVID-19 pandemic and concluded they have escalated stress levels among university students. These alterations have contributed to a surge in negative emotional symptoms, potentially escalating into more severe mental health challenges [6]. Among some other results from this meta-analysis, we highlight the most relevant as follows: higher prevalence rates of anxiety, depression, and stress have been reported, which were exacerbated during the lockdown and the changes in education format (the switch to online classes). The prevalence of anxiety and depression among students was notably higher than in the general population, with European students demonstrating some of the highest rates. Stress was also significantly elevated, though rates varied by country and region. Factors contributing to these findings included the severity and duration of lockdown measures, social isolation, and reduced social contact.

The preventive measures adopted by the Romanian government also included changes in education format in medical universities. Medical education was switched to an online format in 2020, gradually moving to a hybrid form of education with courses and large gatherings taking place online, but with physical clinical rotations. Gradually, a full on-site format was resumed by the end of 2021, although very strict social and hygiene regulations persisted through 2022. Most medical universities in Romania adopted a mandatory vaccination policy, both for students and personnel, with exceptions for medical reasons only.

The landscape of medical education was reshaped by the outbreak of the COVID-19 pandemic. Medical students faced unique challenges, with disruptions to clinical rotations, limited access to hands-on training, and the psychological impact of witnessing the devastating effects of the virus on patients and healthcare systems. The pandemic has heightened the importance of examining mental health implications for medical students and exploring strategies to mitigate the emotional toll of this unprecedented crisis. A study by Mahardani et al. delves into the impact of the COVID-19 pandemic on medical students’ stress levels and academic achievement. The research highlights the heightened stress levels experienced by medical students during online learning, with a majority attributing their stress to academic-related stressors (ARS) [7].

Stress is a significant risk factor for the development and continuation of alcohol abuse [8]. The pandemic has affected several populations, with reported increases in alcohol consumption [9,10]. The respondents in a survey also noted that they consumed more alcohol when experiencing higher levels of anxiety or sadness [10]. A review by Boden and Fergusson found a significant association between alcohol use disorders and depression, with each disorder doubling the risk of the other [11].

The closure of campuses and the return of students to their family homes may not have helped with unhealthy coping mechanisms such as smoking and drinking. Also, rising levels of anxiety and stress could lead to more frequent use of unhealthy but socially encouraged coping mechanisms [12].

The main findings of a study conducted in Romania on medical students in the early days of the pandemic showed a high level of stress, burnout, anxiety, and fear related to COVID-19 [13]. Female students and those with a history of mental health issues were particularly affected. The use of psychoactive substances, such as tobacco and alcohol, was also prevalent among medical students, with concerns raised about the potential for substance abuse during times of stress. The above-mentioned study highlighted the importance of addressing mental health issues among medical students during the pandemic, particularly given their potential exposure to COVID-19 as part of their studies.

Another study on this topic examined the relationship between psychological distress, social support, and changes in alcohol use following the COVID-19-related closures of universities [14]. The authors surveyed undergraduate students about their alcohol use before and after the closures caused by the restrictions, as well as their levels of distress and support. The study findings indicated that students faced with elevated distress levels and limited social support were more inclined to report increased alcohol consumption during the pandemic.

### Aims and Research Questions

The aim of this article is to provide valuable insights into how the COVID-19 pandemic has impacted academic stress, depression, and alcohol consumption among medical students. We seek to understand the challenges faced by medical students during the pandemic, which has brought significant changes to daily life. Specifically, we will explore how the pandemic has affected academic stress levels and alcohol consumption among this student population. Our goal is to contribute to a better understanding of how COVID-19 has influenced the mental and physical health of medical students, with the hope that our findings can inform future research and policy decisions. The research questions proposed for this study are:

1. How has the COVID-19 pandemic impacted the levels of academic stress and depression among medical students, and how does this compare to pre-pandemic levels? Has there been a change in academic stress levels and alcohol use patterns between 2018 (before COVID-19) and 2022 (after the loosening of restrictions). If so, how significant is the change?
2. Has the COVID-19 pandemic led to an increase in alcohol consumption among medical students, and if so, what factors are associated with this increase?
3. Are there specific demographic or academic factors that are associated with increased stress or alcohol consumption among medical students during the COVID-19 pandemic?
4. To what extent is fear of COVID-19 associated with depression, academic stress and alcohol consumption among medical students?

Following the research questions, our research hypotheses are as follows: we expect that the COVID-19 pandemic likely resulted in elevated levels of academic stress and depression among medical students in 2022 when compared to the pre-pandemic year of 2018, potentially leading to shifts in alcohol consumption habits [8,9,10]. We expect that the heightened stress and feelings of isolation due to pandemic-related restrictions led to the adoption of alcohol as a coping mechanism [10]. Demographic and academic factors, such as gender, academic year, and geographical background, are expected to play a role in shaping variations in stress levels and alcohol consumption patterns among medical students throughout the pandemic [13]. Fear of COVID-19 is likely to be intertwined with higher rates of depression, increased academic stress, and greater alcohol use, underscoring its significant impact on the mental well-being of medical students [13].

## 2. Materials and Methods

### 2.1. Participants and Procedure

Two cross-sectional and anonymous online surveys were conducted at the University of Medicine and Pharmacy “Iuliu Hatieganu” in Romania among the medical students. The surveys were elaborated upon with the support of the University Counselling Centre and were targeted at students from the Faculty of Medicine who were studying for a medical degree across all six years of study.

We conducted our first cross-sectional study in 2018 to investigate the stress, depression, and alcohol and drugs used by medical students; the second study in 2022 was conducted to determine the impact of the pandemic and online education on the mental health status of the students. Overall, 1061 students completed the online survey (781 in 2018 and 280 in 2022). The study was approved by the ethics committee of the University of Medicine and Pharmacy “Iuliu Hațieganu” Cluj Napoca.

The necessary data for the study were collected through a survey based on an anonymously administered online questionnaire, in which participants willingly granted their consent for data processing and publication. This approach allowed participants to voluntarily engage with the questionnaire from the convenience of their own homes, maintaining the confidentiality of their identities. The distribution of the questionnaire to the target audience occurred in the online environment, specifically through closed groups of students associated with the abovementioned academic years on the social media platform Facebook, and, in 2022, also on Microsoft Teams (the platform used by the university for online teaching).

The first survey was made available for completion during the academic year 2017–2018, specifically within the examination session periods. As for the second survey, the questionnaire was accessible for completion during the academic year 2021–2022, in the latter part of the pandemic, during a period characterized by partially relaxed societal restrictions, but very strict health regulations within the university. We chose the instruments included in our survey based on their psychometric properties and clinical relevance.

### 2.2. Questionnaire

The online questionnaire was divided into multiple sections with the purpose of facilitating a systematic collection of data.

#### 2.2.1. Demographic Information

The first section was dedicated to demographic information. We designed the demographic section to obtain the general characteristics of medical students, which included: gender; educational level of parents (high school or higher education); financial status; the current year in the medical school (pre-clinical or clinical, as the first three years are pre-clinical and the last three are clinical); modalities of financing the studies (financed by the government or paid for by the student in the form of an annual tax); and admission to the medical school (first attempt or after more than one try). Financial status was assessed as follows: (1) low: barely sufficient to provide basic family needs; (2) medium: sufficient to provide basic family needs; (3) good: can afford everything needed for a normal life; and (4) excellent: can consume without any restriction. We also asked the participants whether they were living in an urban or a rural area.

#### 2.2.2. Beck Depression Inventory

The second section aimed to assess depression using the Beck Depression Inventory-Romanian version (BDI II) as an assessment tool [15]. The Beck Depression Inventory (BDI-II) is one of the most widely used self-report measures of depression in both research and clinical practice, with high validity and good psychometric properties [14]. For the Romanian version, Cronbach Alpha is 0.89 [15]. The questionnaire consists of 21 items, and answers are rated on a four-point scale (0 = low, 3 = high). The total score ranges from 0 to 63. For a clinical investigation of depression, a score from 0–13 indicates that a person is not depressed, 14–19 indicates mild–moderate depression, 20–28 indicates moderate–severe depression and 29–63 indicates severe depression [16,17].

#### 2.2.3. Medical Student Stressor Questionnaire (MSSQ)

The third section aimed to quantify the stress factors implicated in medical students’ lives. For this assessment, the Romanian version of the Medical Student Stressor Questionnaire (MSSQ) was used [18], a validated tool for analyzing stressors in the medical academic environment [19,20]. The total Cronbach’s alpha value of the Romanian version of MSSQ was 0.884. The reliability coefficients of the stressor groups range from 0.704 to 0.902 [18]. The MSSQ contains 20 items, each representing a possible stress factor for medical students identified in the literature, categorized into six domains: academic stressors (ARS), intrapersonal and interpersonal stressors (IRS), stress related to education and study (TLRS), social stressors (SRS), stress related to motivation and desires (DRS), and stress related to group activities (GARS). Participants were asked to evaluate each stressor based on intensity: “no stress”, “mild stress”, “moderate stress”, “high stress”, and “very high stress”. For preclinical students who had not had extensive patient contact, there was an option “I have not had contact with patients” concerning items related to patient interaction. After calculating the score, there were four result categories: a score between 0–1 indicated mild stress, 1–2 indicated moderate stress, 2–3 indicated high stress, and 3–4 indicated severe stress [18].

#### 2.2.4. Alcohol Consumption

The fourth section of the questionnaire addressed alcohol consumption. It included questions related to the participant’s status as a consumer and their perception of alcohol consumption from the beginning of their university studies or during periods of stress. Furthermore, the risk of alcohol abuse was assessed using the Alcohol Use Disorders Identification Test (AUDIT) [21], developed by the World Health Organization for easily screening alcohol abuse or excessive consumption. This test has proven its utility in evaluating such behavior among students [22]. The AUDIT questionnaire contains 10 items that assess three aspects related to alcohol consumption: excessive quantity, symptoms of dependence, and negative effects.

The subsequent section assessed smoking among medical students, focusing on their smoking status, frequency of smoking, number of cigarettes consumed, and any changes in smoking habits since the beginning of their higher education.

The questionnaire also aimed to collect data on the prevalence of illicit psychoactive substance use in accordance with Romanian legislation. The first question in this section addressed the consumption of illicit psychoactive substances such as marijuana, hashish, amphetamines, synthetic drugs, MDMA (Ecstasy), LSD (lysergic acid diethylamide), and cocaine. These substances were chosen based on international studies regarding substance use among medical students [23] and their popularity in mass media and youth social circles. Concerning frequency, each substance mentioned was evaluated using the following consumption parameters: “in the past week”, “in the past month”, “in the past 6 months”, “in the past year”, and “never”. Respondents were not provided with the option to indicate other substances they may have used to avoid inclusion errors, omissions, or nomenclature issues.

#### 2.2.5. COVID-19-Related Anxiety and Fear

In 2022, we added a new section to the survey regarding the experience of anxiety and fear in relationships with the COVID-19 pandemic. We used two questionnaires to assess COVID-19-related anxiety and fear that were validated in a previous study [13]. The first questionnaire has 12 items, was adapted after Ho et al. [24] and assessed the participants’ opinion via a 4-point Likert scale (0—definitely false, 3—definitely true). The second questionnaire was adapted after Ahorsu et al. [25], has 7 items, and the answers are given via a 5-point Likert scale (1—strongly disagree, 5—strongly agree). For the Romanian version, the Cronbach Alpha is 0.82 and 0.84 [13]. Students were asked to answer how they feel in the present regarding COVID-19 and how they remembered the feelings and experiences they had in 2020. We also asked the students if they had had COVID-19, if they had lost a family member to COVID-19, and if they were vaccinated.

### 2.3. Statistical Analysis

The statistical analysis was performed using SPSS, version 26 for Windows. Categorical variables were reported by frequency and percentage, and continuous variables were illustrated by the mean and standard deviation. The normality of the distributions of the data was first assessed by Kolgorov–Smirnov’s test. Because the data was not normally distributed, between-group comparisons of continuous variables were performed using the Mann–Whitney U-test and Wilcoxon Signed Rank test. Correlation analysis was performed using Pearson’s correlation coefficient. The results were considered statistically significant if the *p*-value was less than 0.05.

A multiple linear regression was calculated to predict depressive symptoms (BDI sum score) based on socioeconomic and demographic variables (gender, financial situation, marital status, residence, financing of the studies, attempts to enter the medical school, clinical/nonclinical year). Categorical variables with more than two categories were recoded and dichotomized (financial situation, marital status, clinical/nonclinical year). All predictor variables were entered simultaneously. A further 6 linear regressions were calculated to predict the stress associated with medical education (one for each subscale of the MSSQ and one linear regression for alcohol consumption (AUDIT score). We used the same predictors as the first regression.

## 3. Results

### 3.1. Demographic Data

The mean age was 22.46 years (±2.07) in 2018 and 22.51years (±2.79) in 2022. In 2018, 49.4% of the participants were single and only 2.2% were married (48.4% and 2.9% in 2022). Most of the students had parents who graduated from higher education, mothers (67% and 66.1%) and fathers (62.20% and 60.7%), and among these, 12% and 8.6%, respectively, of the parents were physicians. The majority of the participants were living in a city (85% and 85.7%) and were in a pre-clinical year (67.1% and 63.9%). Most of the students had a good or excellent financial situation. In 2018, 24.2% of the students were paying for their education (17.5% in 2022) and around 80% were admitted into medical school upon the first examination attempt. The socio-demographic profile of the participants is presented in Table 1.

### 3.2. Stress-Related Data

Degree of stress and mean stress score across all six domains are represented in Table 2. The highest mean degrees were attributed to academic-related stressors (ARS), followed by group-activity-related stressors (GARS). The mean stress levels for the other four domains were within the range of mild to moderate levels.

Table 3 presents the comparison of stress levels among students, in the 2 years of the survey.

In 2018 students scored significantly higher in the overall domains of academic-related stressors (ARS), interpersonal- and intrapersonal-related stressors (IRS), teaching- and learning-related stressors (TLRS) and group-activity-related stressors (GARS), and in 2022 the students scored higher on social-related stressors (SRS).

### 3.3. Substance-Use-Related Data

Substance use can be characteristics of the participants can be consulted in Table 4. In 2018 the number of students who drank alcohol, smoked cigarettes, or used drugs was 430 (55.1%), 348 (44.6%), and 276 (35.3%) respectively. In 2022 the number of students who drank alcohol, smoked cigarettes, or used drugs was 144 (51.4%), 123 (43.9%), and 82 (29.3%), respectively. The only statistically significance differences were that in 2022, the percentage of students who drank more during the exam session and who had used drugs was lower than in 2018.

Table 5 presents the frequency of drug consummation.

Information about alcohol consumption and risk is presented Table 4, Table 6 and Table 7, covering AUDIT score and category comparisons. In Table 8 we present a summary of the behavioral consequences of alcohol use. There were no differences regarding the AUDIT scoring categories. Although the percentage of students who had behavioral problems because of drinking seems lower in 2022, the differences were not statistically significant.

### 3.4. Depression-Related Data

Mean depression score on BDI was significantly lower in 2022; the difference in the percentage of students who had suicidal ideation is not statistically significant (Table 9). In a comparative analysis between 2018 and 2022 (Table 10), the data reveal a notable shift in the prevalence of depression, with a marked increase in individuals categorized as ‘Not depressed’ from 57.7% to 68.2% and a significant decrease in those with ‘Severe Depression’ from 9.3% to 2.9%, suggesting an overall improvement in mental well-being.

The percentage of individuals currently diagnosed with depression increased from 1.7% to 4.6%; there was a marginal rise in those previously diagnosed, from 5.4% to 7.1% (Table 11). In the 2022 cohort, a greater proportion of students received antidepressant prescriptions from a psychiatrist (Table 12).

### 3.5. Correlations and Regressions

We found a significant correlation between depression and alcohol use assessed with AUDIT and between depression and all domains of academic stress (ARS academic-related stressor, IRS interpersonal- and intrapersonal-related stressor, TLRS teaching- and learning-related stressor, SRS social-related stressor, DRS drive- and desire-related stressor, GARS group activity-related stressor).

Alcohol use assessed with AUDIT had a significant correlation with the following domains of academic stress: IRS interpersonal- and intrapersonal-related stressor; TLRS teaching- and learning-related stressor; and DRS drive- and desire-related stressor. More data are available in Table 13.

An exploratory multiple regression analysis examined predictors of depressive symptomatology (see Table 14). Female gender (*p* = 0.000), having financial problems (*p* = 0.000), paying for the studies (*p* = 0.012) and being in pre-clinical years (*p* = 0.001) significantly predicted higher levels of depressive symptoms. All other predictors were unrelated to depressive symptoms (all *p* > 0.05). Collectively, the model explains approximately 53% (Adjusted R2 = 0.53) of the variance in the outcome, with a highly significant overall model fit (F = 9.417, *p* < 0.001).

An exploratory multiple regression analysis examined predictors of ARS academic-related stressors (see Table 15). Female gender (*p* = 0.000), having financial problems (*p* = 0.001), paying for the studies (*p* = 0.006) and being in pre-clinical years (*p* = 0.000) significantly predicted higher levels of academic-related stressors. All other predictors were unrelated to academic-related stressors (all *p* > 0.05). Altogether, the model accounts for approximately 72% of the variance in the outcome (Adjusted R2 = 0.72), and the overall model fit is highly significant with an F-statistic of 12.72 (*p* < 0.001).

Table 16 shows an exploratory multiple regression analysis regarding the predictors of IRS interpersonal- and intrapersonal-related stressors. Female gender (*p* = 0.000) and having financial problems (*p* = 0.042) significantly predicted higher levels of IRS interpersonal- and intrapersonal-related stressors. All other predictors were unrelated to IRS interpersonal- and intrapersonal-related stressors (all *p* > 0.05). The overall model fit was R2 = 0.26 (adjusted R2 = 0.19).

Regarding the teaching- and learning-related stressor (see Table 17), female gender (*p* = 0.002) and having financial problems (*p* = 0.009) significantly predicted higher levels of TLRS. All other predictors were unrelated to TLRS teaching- and learning-related stressors (all *p* > 0.05). The overall model fit was R2 = 0.25 (adjusted R2 = 0.19).

An exploratory multiple regression analysis examined predictors of GARS group-activity-related stressors (see Table 18). Female gender (*p* = 0.000), having financial problems (*p* = 0.014) and paying for the studies (*p* = 0.005) significantly predicted higher levels of GARS group-activity-related stressors. All other predictors were unrelated to GARS group-activity-related stressors (all *p* > 0.05). This model explains around 74% of the variance in the dependent variable, as indicated by the adjusted R2 value of 0.74 and possesses a highly significant overall model fit with an F-statistic of 9.925 (*p* < 0.001).

The multiple regression analysis examining predictors of the social-related stressors and the drive- and desire-related stressors showed that all the predictors were unrelated.

In the AUDIT regression analysis (see Table 19), male gender (*p* = 0.000), and living at an urban residence (*p* = 0.008) significantly predicted higher levels of alcohol use. Other variables like financial situation, marital status (single), paying for studies, and being in the pre-clinical stage showed no statistically significant relationships (all *p* > 0.05). The overall model accounts for approximately 43% of the variance in the outcome.

In comparing students who had lost a family member to those who had not (see Table 20), the group experiencing a family loss showed a higher average BDI score of 15.26, compared to 10.77 in the latter group; this difference was statistically significant with a *p*-value of 0.002. While past fears related to COVID-19 in 2020 did not differ significantly between the two groups (*p* > 0.05), there was a notable difference in the level of anxiety felt at the moment of the survey, with the group that had experienced a family loss showing a higher mean score of 10.26 against 8.54 in the other group, and this was statistically significant with a *p*-value of 0.03. However, other measurements related to COVID-19 fears and anxieties either in the past or at the survey’s moment did not demonstrate significant disparities between the two groups.

## 4. Discussion

Our study examined demographic factors, stress levels, substance use, and mental health among medical students in 2018 and 2022. While demographic characteristics remained relatively consistent over the years, academic-related stressors were the most significant stressors, with a surprising decrease in their intensity noted in 2022. Substance use patterns, including alcohol consumption and drug use, showed little change between the two years. A meta-analysis of studies on depression in university students before the pandemic found that the overall prevalence of depressive symptoms was 24.4% [26].

Our results showed that depression scores in 2022 were significantly lower than in 2018, pointing to improved mental well-being, although the prevalence of diagnosed depression increased. Correlations were found between depression, alcohol use, and various stress domains. Female gender, financial issues, and being in pre-clinical years were associated with higher stress and depression levels. These findings underscore the importance of continued mental health support and resources for medical students, particularly in managing academic stress.

Understanding the intricacies of these factors is pivotal in designing effective and tailored mental health interventions for medical students. By recognizing the unique stressors and challenges faced by future physicians, educators and healthcare institutions can collaboratively work towards creating a nurturing environment that fosters not only academic excellence but also the emotional resilience and mental well-being of these future healthcare professionals.

One of the main observations is that academic-related stressors (ARS) consistently had the highest mean stress scores over the years, indicating that medical curriculum demands and academic pressures significantly affect students, regardless of a pandemic context.

Elevated levels of stress, anxiety, and depression among medical students are frequently documented in the existing literature both before and after the pandemic [27,28,29,30,31] as well as during the pandemic [7,32,33,34]. However, there are limited data available that directly compare these two situations. The mean depression scores decreased from 2018 to 2022, indicating a potential improvement in mental well-being among medical students. A higher percentage of students had been diagnosed with depression by a psychiatrist in 2022, and more students were prescribed antidepressants.

Paradoxically, while the pandemic introduced new stressors [35,36], such as health and family concerns, social isolation, and disruptions in everyday life, it also prompted changes in the medical education system. The progress in remote learning options and resources and telemedicine allowed some students more control over their learning schedules [37]. This flexibility might have alleviated some stressors associated with the traditional medical learning environment. Also, support services for mental health and well-being might not have been as readily available or prioritized before the pandemic and students may have had fewer resources to cope with stress and seek help. Therefore, the pandemic might have forced educators and institutions to adapt quickly, leading to more awareness of students’ well-being and the implementation of support mechanisms. As a result of the pandemic, educators and institutions were forced to adapt quickly, leading to a greater focus on students’ well-being and the implementation of support systems. Additionally, the pandemic unintentionally led to a notable increase in awareness about mental health [38], largely due to the profound struggles individuals faced during quarantine. A possible future direction in the research of this topic is to look at how university counselling services may have changed in the context of the pandemic and how that change affected students’ mental health, and the need for specific support for medical faculties [39,40].

An interesting observation is that in 2022, students reported higher stress compared with 2018 in only one stress domain, that being the social-related stressor (SRS). We can suppose that after a long period of quarantine and remote learning, being back in a social educational environment might pose some challenges to the student; this indirectly suggests that students might find it difficult to engage in social and community activities after experiencing prolonged isolation.

Another important outcome of our study is the importance of gender. Being female, having financial problems, and being in pre-clinical years were predictors of higher academic-related stress and depressive symptoms. Similar findings have been suggested in the literature [25,27,31], emphasizing the need for gender-specific mental health support and interventions.

Financial problems and the correlation with stress and depression may be rooted in how universities are funded. In Romania, there is an admission exam for medical school; some positions are subsidized by the government and education is free for the student and some fees are paid for by the student with an annual tax. The distribution to one of the subsidized or the paid positions is based on the academic performance of the student and changes every year, meaning that from one year to the next, if a student does not perform, they might lose their subsidized position and start to pay a tax.

The percentage of students who consumed alcohol remained relatively stable. The AUDIT scoring did not show significant changes between 2018 and 2022, suggesting a stagnation in alcohol use without a clear increase in dependency, corroborating data from a previously published study [34]. However, this comes as a surprise, since notable studies correlate stress and alcohol use. In our research, this was not the case [10,11]. Being male and residing in an urban area were predictors of higher alcohol use, and this suggests the need for targeted alcohol awareness campaigns and support for male students in urban settings.

Our results offer some valuable contributions to several Global Goals of the 2030 Agenda for Sustainable Development [41]. First, by identifying the prevalence of depression among medical students and potential risk factors, the study supports good health and well-being by providing insights into mental health issues, paving the way for improved well-being within the healthcare workforce. Furthermore, the study indirectly contributes to the goal of quality education by emphasizing the need for supportive learning environments and the well-being of students as integral to education. Gender disparities in mental health, with female students reporting higher levels of depression, highlight the importance of addressing gender equality within medical education. The impact of socioeconomic factors on mental health underscores the need to reduce inequalities in access to education and healthcare, aligning with the goal to reduce inequality. Lastly, these findings can catalyze partnerships among educational institutions, healthcare providers, and policymakers to collectively work toward these goals and foster sustainable development, as outlined in the objective of creating partnerships for the goals.

### Limitations

Our study has several limitations. First, the data collection relied on self-report questionnaires, which can be subject to bias. Participants may underreport or overreport certain behaviors or feelings due to social desirability bias or memory recall issues. Additionally, the response rate for the 2022 survey was lower than that of the 2018 survey; this discrepancy could be explained by a non-response bias, as those who chose not to participate may have different experiences or characteristics from those who did respond. Furthermore, findings from this study may not be generalizable to medical students in other countries or cultural contexts. The experiences and coping mechanisms of medical students can vary widely based on cultural, educational, and healthcare system differences. Additionally, while the study acknowledges various demographic and academic factors that may influence stress levels and alcohol consumption, there may be other unmeasured variables unaccounted for, such as personal life events or external stressors, that could impact the results but are not accounted for in the analysis. Lastly, the surveys were conducted at different points in the academic year, and the pandemic situation evolved over time. This could introduce variability in responses based on the timing of data collection.

## 5. Conclusions

In conclusion, our research highlights the dynamic nature of stress, substance use, and mental health among medical students. The findings suggest the importance of tailored interventions and support systems to address the unique challenges faced by different groups of students, such as gender-specific mental health programs and financial assistance for those in need. Additionally, the decrease in substance use and depression scores over time may indicate the effectiveness of awareness and support initiatives within the medical education community. Further research could explore the specific factors contributing to these changes and their long-term implications for the wellbeing of medical students.

## Figures and Tables

**Table 1:** Socio-demographic characteristics of the participants.

	2018 (N = 781)	2022 (N = 280)
**Age (mean ± SD) years**	22.46 (±2.07)	22.51 (±2.79)
**Sex**		
Male	624 (20.0%)	157 (20.1%)
Female	224 (80%)	56 (79.9%)
**Marital status**		
Single	386(49.4%)	132 (47.1%)
In a relationship	378 (48.4%)	140 (50%)
Married	17 (2.2%)	8 (2.9%)
**Residence**		
Urban	664 (85%)	240 (85.7%)
Rural	117 (15%)	40 (14.3%)
**Financial situation**		
Low	21 (2.7%	2 (0.7%)
Medium	123 (15.7%)	39 (13.9%)
Good	564 (72.2%)	206 (73.6%)
Excellent	73 (9.3%)	33 (11.8%)
**Year of the studies**		
Pre-Clinical	420 (67.1%)	206 (63.9%)
Clinical	278 (32.9%)	157 (36.1%)
**Level of education of the mother**		
High school	258 (33.00%)	95 (33.90%)
Higher education	523 (67.00%)	185 (66.10%)
**Level of education of the father**		
High school	295 (37.80%)	110 (39.30%)
Higher education	486 (62.20%)	170 (60.70%)
**One or both parents physicians**		
Yes	94 (12.00%)	24 (8.60%)
No	687 (88.00%)	256 (91.40%)
**Financing of the studies**		
Free	592 (75.80%)	231 (82.50%)
With tax	189 (24.20%)	49 (17.50%)
**Admission to the medical school on the first try**		
Yes	629 (80.50%)	222 (79.30%)
No	152 (19.50%)	58 (20.70%)

**Table 2:** Degree of stress and mean stress score across all six domains.

Domains of Stressors		Mild, N (%)	Moderate, n (%)	High, n (%)	Severe, n (%)	Mean Score	Grade
ARS	2018	27 (3.5%)	102 (13.1%)	260 (33.3%)	392 (50.2%)	2.97 (±0.84)	High
	2022	33 (11.8%)	87 (31.1%)	85 (30.4%)	75 (26.8%)	2.33 (±1.01)	High
IRS	2018	350 (44.8%)	187 (23.9%)	143 (18.3%)	101 (12.9%)	1.53 (±1.26)	Moderate
	2022	157 (56.1%)	63 (22.5%)	41 (14.6%)	19 (6.8%)	1.25 (±1.25)	Moderate
TLRS	2018	88 (11.3%)	251 (32.1%)	279 (35.7%)	163 (20.9%)	2.3662 (±0.97)	High
	2022	139 (49.6%)	62 (22.1%)	35 (12.5%)	44 (15.7%)	1.7 (±1.46)	Moderate
SRS	2018	423 (54.2%)	215 (27.5%)	98 (12.5%)	45 (5.8%)	1.15 (±1.12)	Moderate
	2022	97 (34.6%)	91 (32.5%)	54 (19.3%)	38 (13.6%)	1.80 (±1.32)	Moderate
DRS	2018	521 (66.7%)	145 (18.6%)	52 (6.7%)	63 (8.1%)	1.00 (±1.19)	Moderate
	2022	148 (52.9%)	81 (28.9%)	28 (10.0%)	23 (8.2%)	1.34 (±1.23)	Moderate
GARS	2018	143 (18.3%)	196 (25.1%)	233 (29.8%)	209 (26.8%)	2.30 (±1.12)	High
	2022	90 (32.1%)	55 (19.6%)	50 (17.9%)	85 (30.4%)	2.32 (±1.66)	High
Total stress	2018	92 (11.8%)	345 (44.2%)	263 (33.7%)	81 (10.4%)	1.88 (±0.74)	Moderate
	2022	67 (24.2%)	94 (33.9%)	81 (29.2%)	35 (12.6%)	1.86 (±0.82)	Moderate

ARS: academic-related stressor, IRS: interpersonal- and intrapersonal-related stressor, TLRS: teaching- and learning-related stressor, SRS: social-related stressor, DRS: drive- and desire-related stressor, GARS: group-activity-related stressor.

**Table 3:** Stress levels comparisons.

Domains of Stressors		No/Low Stress		High Stress		Chi Square	*P*	Cramer’s V *p*	Odds Ratio	95% Confidence Interval	
		N	%	N	%					Lower	Upper
ARS	2018	129	16.5%	652	83.5%	79.61	0.000	0.000	0.264	0.195	0.357
	2022	120	42.9%	160	57.1%						
IRS	2018	537	68.8%	244	31.2%	9.70	0.002	0.002	0.600	0.435	0.829
	2022	220	78.6%	60	21.4%						
TLRS	2018	339	43.4%	442	56.6%	69.81	0.000	0.000	0.291	0.216	0.392
	2022	203	72.5%	77	27.5%						
SRS	2018	638	81.7%	143	18.3%	23.01	0.000	0.000	2.113	1.551	2.880
	2022	190	67.9%	90	32.1%						
DRS	2018	666	85.3%	115	14.7%	1.21	0.270	0.270	1.288	0.852	1.772
	2022	231	82.5%	49	17.5%						
GARS	2018	339	43.4%	442	56.6%	28.97	0.000	0.000	0.467	0.353	0.618
	2022	174	62.1%	106	37.9%						
Total stress	2018	456	58.4%	325	41.6%	0.024	0.877	0.877	1.022	0.775	1.348
	2022	162	57.9%	118	42.1%						

ARS: academic-related stressor, IRS: interpersonal- and intrapersonal-related stressor, TLRS: teaching- and learning-related stressor, SRS: social-related stressor, DRS: drive- and desire-related stressor, GARS: group-activity-related stressor.

**Table 4:** Smoking, alcohol, and drug consumption.

	2018 (N = 781)	2022 (N = 280)	Chi Square *p*	Cramer’s V *p*	Odds Ratio	95% Confidence Interval	
						Lower	Upper
**Drinking alcohol**							
Yes	430 (55.1%)	144 (51.4%)	*p* > 0.05	*p* > 0.05	0.864	0.657	1.136
No	351 (44.9%)	136 (48.6%)					
**Do you drink more alcohol when you are stressed (during the exam session)**							
Yes	81 (18.8%	17 (6.1%)	*p* < 0.000	*p* < 0.000	0.279	0.161	0.481
No	349 (81.2%)	263 (93.9%)					
**How often do you smoke**							
Never	433 (55.4%)	157 (56.1%)	*p* > 0.05	*p* > 0.05			
Occasionally	174 (22.3%)	54 (19.3%)					
Daily	174 (22.3%	69 (24.6%)					
Average number of cigarettes/days	9.3063 (±6.64660)	10.2209 (±7.43822)	*p* > 0.05 *			−2.63	0.08
**Energy drinks**							
Yes	216 (27.7%)	88 (31.4%)	*p* > 0.05	*p* > 0.05	1.199	0.981	1.614
No	565 (72.3%)	192 (68.6%)					
**Did you ever use drugs**							
Yes	276 (35.3%)	82 (29.3%)	*p* < 0.05	*p* < 0.05	0.775	0.576	1.042
No	505 (64.7%)	198 (70.7%)					

* *t*-test.

**Table 5:** Frequency of drug consummation.

How Often Do You Use Drugs				
	2018 (N = 781)	2022 (N = 280)	Chi Square *p*	Cramer’s V *p*
**Marijuana**				
Never	505 (64.7%	192 (68.6%)	*p* < 0.006	*p* < 0.006
In the last years	101 (12.9%)	52 (18.6%)		
In the last 6 months	99 (12.7%)	21 (7.5%)		
In the last month	41 (5.2%)	8 (2.9%)		
In the last week	35 (4.5%)	7 (2.5%)		
**Amphetamine**				
Never	726 (93.0%)	265 (94.6%)	*p* > 0.05	*p* > 0.05
In the last years	20 (2.6%)	10 (3.6%)		
In the last 6 months	9 (1.2%)	3 (1.1%)		
In the last month	12 (1.5%)	1 (0.4%)		
In the last week	14 (1.8%)	1 (0.4%)		
**Synthetic drugs**				
Never	725 (92.8%)	275 (98.2%)	*p* < 0.02	*p* < 0.02
In the last years	23 (2.9%)	3 (1.1%)		
In the last 6 months	8 (1.0%)	1 (0.4%)		
In the last month	13 (1.7%)	0 (0.0%)		
In the last week	12 (1.5%)	1 (0.4%)		
**MDMA**				
Never	715 (91.5%)	269 (96.1%)	*p* < 0.05	*p* < 0.05
In the last years	20 (2.6%)	7 (2.5%)		
In the last 6 months	16 (2.0%)	2 (0.7%)		
In the last month	18 (2.3%	1 (0.4%)		
In the last week	12 (1.5%)	1 (0.4%)		
**LSD**				
Never	735 (94.1%)	274 (97.9%)	*p* > 0.05	*p* > 0.05
In the last years	9 (1.2%)	3 (1.1%)		
In the last 6 months	12 (1.5%)	2 (0.7%)		
In the last month	13 (1.7%)	1 (0.4%)		
In the last week	12 (1.5%)	0 (0.0%)		

**Table 6:** AUDIT scoring (N = 574 students who drink alcohol).

Year	N	Mean (SD)	Mann–Whitney P
2018	430	5.05 (3.96)	*p* > 0.05
2022	144	4.86 (4.35)	

**Table 7:** Audit categories (N = 574 students who drink alcohol).

Year	Low Risk		Increasing Risk		Higher Risk		Possible Dependence		Chi Square *p*	Cramer’s V *p*
	N	%	N	%	N	%	N	%		
2018	351	81.60%	67	15.60%	7	1.60%	5	1.20%	>0.05	>0.05
2022	117	81.3%	24	16.7%	1	0.70%	2	1.4%		

**Table 8:** Behavioral consequences of alcohol use.

		Never N (%)	Many Years Ago N (%)	At Least Once in the Last Year N (%)	At Least Once in the Last 6 Months N (%)	At Least Once in the Last Month N (%)	More than Once in the Last 6 Months N (%)	Chi Square *p*	Cramer’s V *p*
Had a hangover	2018	58 (13.5%)	56 (13.0%)	131 (30.5%)	92 (21.4%)	48 (11.2%)	45 (10.5%)	>0.05	>0.05
	2022	20 (13.9%)	31 (21.5%)	28 (19.4%)	25 (17.4%)	18 (12.5%)	22 (15.3%)		
Gotten nauseated and vomited from drinking	2018	78 (18.1%)	102 (23.7%)	130 (30.2%)	74 (17.2%)	19 (4.4%)	27 (6.3%)	>0.05	>0.05
	2022	33 (22.9%)	39 (27.1%)	32 (22.2%)	22 (15.3%)	6 (4.2%)	12 (8.3%)		
Binge drinking	2018	193 (44.9%)	84 (19.5%)	70 (16.3%)	37 (8.6%)	15 (3.5%)	31 (7.2%)	>0.05	>0.05
	2022	73 (50.7%)	35 (24.3%)	10 (6.9%)	11 (7.6%)	7 (4.9%)	8 (5.6%)		
Gotten in a fight or argument after drinking	2018	389 (90.5%)	25 (5.8%)	6 (1.4%)	7 (1.6%)	3 (0.7%)	389 (90.5%)	>0.05	>0.05
	2022	135 (93.8%)	7 (4.9%)	1 (0.7%)	1 (0.7%)	0 (0.0%)	135 (93.8%)		
Missed a class after having several drinks	2018	321 (74.7%)	39 (9.1%)	31 (7.2%)	21 (4.9%)	8 (1.9%)	10 (2.3%)	>0.05	>0.05
	2022	121 (84.0%)	10 (6.9%)	4 (2.8%)	3 (2.1%)	3 (2.1%)	3 (2.1%)		
Come to class after having several drinks	2018	297 (69.1%)	42 (9.8%	45 (10.5%)	25 (5.8%)	12 (2.8%)	297 (69.1%)	>0.05	>0.05
	2022	110 (76.4%)	20 (13.9%	5 (3.5%)	4 (2.8%)	4 (2.8%)	110 (76.4%)		
Driven a car after having several drinks	2018	387 (90.0%)	21 (4.9%)	8 (1.9%)	5 (1.2%)	5 (1.2%)	4 (0.9%)	>0.05	>0.05
	2022	135 (93.8%)	5 (3.5%)	0 (0.0%)	4 (2.8%)	0 (0.0%)	0 (0.0%)		

**Table 9:** Depression average BDI score.

**BDI Mean Score**	**Mean Score (±SD)**	**Mann–Whitney U**				
2018	13.81 (±9.35)	*p* < 0.001				
2022	11.56 (±8.15)					
**Suicidal Ideation Yes (item 9)**	**N (%)**	**Chi Square**	**Cramer’s V *p***	**Odds Ratio**	**95% Confidence** **interval**	
					**Lower**	**Upper**
2018	138 (17.6%)	*p* > 0.05	*p* > 0.05	0.686	0.521	0.903
2022	40 (14.28%)					

**Table 10:** Depression categories comparison.

	Not Depressed, N (%)	Mild-Moderate Depression, N (%)	Moderate Severe Depression, N (%)	Severe Depression, N (%)	Chi Square	Cramer’s V *p*
2018	451 (57.7%)	136 (17.4%)	121 (15.5%)	73 (9.3%)	*p* < 0.001	*p* < 0.001
2022	191 (68.2%)	41 (14.6%)	40 (14.3%)	8 (2.9%)		

**Table 11:** Have you ever been diagnosed with depression by a psychiatrist?

	No, N (%)	Yes, I am Currently Diagnosed with Depression, N (%)	Yes, I was Diagnosed with Depression in the Past, N (%)	Chi Square	Cramer’s V *p*
2018	726 (93.0%)	13 (1.7%)	42 (5.4%)	*p* < 0.01	*p* < 0.01
2022	247 (88.2%)	13 (4.6%)	20 (7.1%)		

**Table 12:** Have you ever been prescribed antidepressants?

	No, N (%)	Yes, I am Currently on Antidepressant Treatment, N (%)	Yes, in the Past, N (%)	Chi Square	Cramer’s V *p*
2018	747 (95.6%	9 (1.2%)	25 (3.2%)	*p* < 0.01	*p* < 0.01
2022	254 (90.7%)	6 (2.1%)	20 (7.1%)		

**Table 13:** Correlations.

	BDI	Audit	ARS	IRS	TLR	SRS	DRS	GARS
BDI	1	0.265 **	0.381 **	0.263 **	0.388 **	0.192 **	0.292 **	0.390 **
AUDIT	0.265 **	1	0.023	0.087 *	0.146 **	−0.066	0.075 *	−0.05

ARS: academic-related stressor, IRS: interpersonal- and intrapersonal-related stressor, TLRS: teaching- and learning-related stressor, SRS: social-related stressor, DRS: drive- and desire-related stressor, GARS: group activity-related stressor. ** Correlation is significant at the 0.01 level (2-tailed). * Correlation is significant at the 0.05 level (2-tailed).

**Table 14:** Linear regression analysis for predictors of depressive symptoms.

	Unstandardized Coefficients		Standardized Coefficients	95.0% Confidence Interval for B		*T*	*p*
	B	Std. Error	Beta	Lower Bound	Upper Bound		
**Gender (female)**	**2.78**	**0.686**	**0.122**	**1.434**	**4.125**	**4.055**	**0.000**
**Financial situation (low)**	**−3.89**	**0.734**	**−0.162**	**−5.331**	**−2.449**	**−5.298**	**0.000**
Marital status (single)	−0.946	0.553	−0.052	−2.03	0.139	−1.711	0.087
Residence (rural)	−1.02	0.777	−0.04	−2.545	0.504	−1.313	0.189
**Paying for the studies**	**1.664**	**0.662**	**0.076**	**0.366**	**2.963**	**2.515**	**0.012**
Second attempt to medical school	0.063	0.693	0.003	−1.298	1.423	0.09	0.928
**Pre-clinical**	**−1.858**	**0.567**	**−0.1**	**−2.971**	**−0.744**	**−3.274**	**0.001**
R2 (Adjusted R2)	0.59 (0.53)						
F	9.417						
P	0.000						

Bold font indicates statistical significance, *p* < 0.05.

**Table 15:** Linear regression analysis for predictors of ARS academic-related stressor.

	Unstandardized Coefficients		Standardized Coefficients	95.0% Confidence Interval for B		*T*	*p*
	B	Std. Error	Beta	Lower Bound	Upper Bound		
**Gender (female)**	**0.501**	**0.07**	**0.215**	**0.365**	**0.638**	**7.2**	**0.000**
**Financial situation (low)**	**−0.256**	**0.075**	**−0.104**	**−0.403**	**−0.11**	**−3.435**	**0.001**
Marital status (single)	0.004	0.056	0.002	−0.107	0.114	0.065	0.948
Residence (rural)	0.055	0.079	0.021	−0.1	0.21	0.693	0.488
**Paying for the** **studies**	**0.187**	**0.067**	**0.084**	**0.055**	**0.319**	**2.781**	**0.006**
Second attempt to medical school	−0.023	0.07	−0.01	−0.161	0.115	−0.329	0.742
**Pre-clinical**	**−0.209**	**0.058**	**−0.11**	**−0.322**	**−0.096**	**−3.622**	**0.000**
R2 (Adjusted R2)	0.78 (0.72)						
F	12.72						
P	0.000						

Bold font indicates statistical significance, *p* < 0.05.

**Table 16:** Linear regression analysis for predictors of IRS interpersonal- and intrapersonal-related stressor.

	Unstandardized Coefficients		Standardized Coefficients	95.0% Confidence Interval for B		*T*	*p*
	B	Std. Error	Beta	Lower Bound	Upper Bound		
**Gender (female)**	**0.342**	**0.097**	**0.108**	**0.151**	**0.533**	**3.508**	**0.000**
**Financial situation (low)**	**−0.213**	**0.104**	**−0.064**	**−0.418**	**−0.008**	**−2.038**	**0.042**
Marital status (single)	−0.081	0.079	−0.032	−0.235	0.074	−1.025	0.305
Residence (rural)	0.115	0.11	0.032	−0.101	0.332	1.045	0.296
Paying for the studies	0.062	0.094	0.021	−0.122	0.247	0.664	0.507
Second attempt to medical school	0.027	0.099	0.008	−0.167	0.22	0.27	0.787
Pre-clinical	0.187	0.081	0.072	0.029	0.346	2.322	0.02
R2 (Adjusted R2)	0.26 (0.19)						
F	3.994						
P	0.000						

Bold font indicates statistical significance, *p* < 0.05.

**Table 17:** Linear regression analysis for predictors of TLRS teaching- and learning-related stressor.

	Unstandardized Coefficients		Standardized Coefficients	95.0% Confidence Interval for B		*T*	*p*
	B	Std. Error	Beta	Lower Bound	Upper Bound		
**Gender (female)**	**0.278**	**0.089**	**0.096**	**0.103**	**0.453**	**3.113**	**0.002**
**Financial situation (low)**	**−0.249**	**0.096**	**−0.081**	**−0.437**	**−0.061**	**−2.6**	**0.009**
Marital status (single)	−0.076	0.072	−0.033	−0.218	0.065	−1.059	0.29
Residence (rural)	0.134	0.101	0.041	−0.065	0.332	1.32	0.187
Paying for the studies	0.138	0.086	0.049	−0.031	0.307	1.602	0.109
Second attempt to medical school	−0.072	0.09	−0.025	−0.249	0.105	−0.795	0.427
Pre-clinical	−0.098	0.074	−0.041	−0.243	0.048	−1.319	0.187
R2 (Adjusted R2)	0.25 (0.19)						
F	3.879						
P	0.000						

Bold font indicates statistical significance, *p* < 0.05.

**Table 18:** Linear regression analysis for predictors of GARS group-activity-related stressor.

	Unstandardized Coefficients		Standardized Coefficients	95.0% Confidence Interval for B		*T*	*P*
	B	Std. Error	Beta	Lower Bound	Upper Bound		
**Gender (female)**	**0.626**	**0.097**	**0.224**	**0.435**	**0.818**	**6.429**	**0.000**
**Financial situation (low)**	**−0.252**	**0.103**	**−0.087**	**−0.454**	**−0.05**	**−2.454**	**0.014**
Marital status (single)	−0.115	0.079	−0.051	−0.269	0.039	−1.461	0.144
Residence (rural)	0.184	0.11	0.059	−0.032	0.401	1.671	0.095
**Paying for the** **studies**	**0.257**	**0.092**	**0.098**	**0.077**	**0.437**	**2.807**	**0.005**
Second attempt to medical school	−0.041	0.1	−0.014	−0.237	0.156	−0.406	0.685
Pre-clinical	0.053	0.084	0.023	−0.112	0.219	0.632	0.527
R2 (Adjusted R2)	0.82 (0.74)						
F	9.925						
P	0.000						

Bold font indicates statistical significance, *p* < 0.05.

**Table 19:** Linear regression analysis for predictors of alcohol use (AUDIT score).

	Unstandardized Coefficients		Standardized Coefficients	95.0% Confidence Interval for B		*T*	*P*
	B	Std. Error	Beta	Lower Bound	Upper Bound		
**Gender (male)**	**−2.043**	**0.375**	**−0.203**	**−2.78**	**−1.307**	**−5.444**	**0.000**
Financial situation (low)	0.073	0.425	0.006	−0.762	0.908	0.172	0.864
marital status (single)	-0.179	0.309	-0.022	−0.786	0.428	−0.58	0.562
**Residence (urban)**	**−1.234**	**0.467**	**−0.098**	**−2.15**	**−0.317**	**−2.643**	**0.008**
Paying for the studies	−0.015	0.382	−0.001	−0.765	0.734	−0.04	0.968
Pre-clinical	−0.07	0.391	−0.007	−0.836	0.697	−0.178	0.859
R2 (Adjusted R2)	(0.52, 0.43)						
F	5.40						
P	0.000						

Bold font indicates statistical significance, *p* < 0.05.

**Table 20:** Comparation of depression, COVID-19-related anxiety and fear between students who had lost a family member due to COVID-19.

	Mean Score (±SD)		*p* for Mann–Whitney U
	Had Lost a Family Member N = 49	Had not Lost a Family Member N = 231	
BDI	15.26 (±9.79)	10.77 (±7.55)	0.002
COVID-19-related fear—in the past in 2020	12.18 (±8.01)	11.22 (±7.21)	>0.05
COVID-19-related fear—in the moment of survey	8.24 (±7.26)	6.73 (±7.19)	>0.05
COVID-19-related anxiety—in the past in 2020	15.10 (±7.19)	14.27 (±6.67)	>0.5
COVID-19-related anxiety—in the moment of survey	10.26 (±5.28)	8.54 (±3.85)	0.03

## Data Availability

The data presented in this study are available on request from the corresponding author.
